# ECG Changes Through Immunosuppressive Therapy Indicate Cardiac Abnormality in Anti-MDA5 Antibody-Positive Clinically Amyopathic Dermatomyositis

**DOI:** 10.3389/fimmu.2021.765140

**Published:** 2022-01-07

**Authors:** Takashi Matsuo, Tsuneo Sasai, Ran Nakashima, Yoshihiro Kuwabara, Eri Toda Kato, Isao Murakami, Hideo Onizawa, Shuji Akizuki, Kosaku Murakami, Motomu Hashimoto, Hajime Yoshifuji, Masao Tanaka, Akio Morinobu, Tsuneyo Mimori

**Affiliations:** ^1^ Department of Rheumatology and Clinical Immunology, Kyoto University, Kyoto, Japan; ^2^ Cancer Control Center, Osaka International Cancer Institute, Osaka, Japan; ^3^ Department of Cardiovascular Medicine, Kyoto University, Kyoto, Japan; ^4^ Department of Advanced Medicine for Rheumatic Diseases, Kyoto University, Kyoto, Japan; ^5^ Department of Clinical Immunology, Graduate School of Medicine, Osaka City University, Osaka, Japan; ^6^ Takeda General Hospital, Kyoto, Japan

**Keywords:** dermatomyositis, interstitial lung disease, anti-melanoma differentiation-associated gene 5 antibody, electrocardiography, T wave

## Abstract

Anti-melanoma differentiation-associated gene 5 (MDA5) antibody, a dermatomyositis (DM)-specific antibody, is strongly associated with interstitial lung disease (ILD). Patients with idiopathic inflammatory myopathy (IIM) who are anti-MDA5 antibody positive [anti-MDA5 (+)] often experience chest symptoms during the active disease phase. These symptoms are primarily explained by respiratory failure; nevertheless, cardiac involvement can also be symptomatic. Thus, the aim of this study was to investigate cardiac involvement in anti-MDA5 (+) DM. A total of 63 patients with IIM who underwent electrocardiography (ECG) and ultrasound cardiography (UCG) during the active disease phase from 2016 to 2021 [anti-MDA5 (+) group, n = 21; anti-MDA5-negative (-) group, n = 42] were enrolled in the study, and their clinical charts were retrospectively reviewed. The ECG and UCG findings were compared between the anti-MDA5 (+) and anti-MDA5 (-) groups. All anti-MDA5 (+) patients had DM with ILD. The anti-MDA5 (+) group showed more frequent skin ulcerations and lower levels of leukocytes, muscle enzymes, and electrolytes (Na, K, Cl, and Ca) than the anti-MDA5 (-) group. According to the ECG findings obtained during the active disease phase, the T wave amplitudes were significantly lower for the anti-MDA5 (+) group than for the anti-MDA5 (-) group (I, II, and V4–6 lead; *p* < 0.01; aVF and V3, p < 0.05). However, the lower amplitudes were restored during the remission phase. Except for the E wave, A wave and Sep e’, the UCG results showed no significant differences between the groups. Four patients with anti-MDA5 (+) DM had many leads with lower T wave and cardiac abnormalities (heart failure, diastolic dysfunction, myocarditis) on and after admission. Though anti-MDA5 (+) patients clinically improved after immunosuppressive therapy, some of their ECG findings did not fully recover in remission phase. In conclusion, anti-MDA5 (+) DM appears to show cardiac involvement (electrical activity and function) during the active phase. Further studies are necessary to clarify the actual cardiac condition and mechanism of these findings in patients with anti-MDA5 (+) DM.

## Introduction

Idiopathic inflammatory myopathy (IIM) is an autoimmune disease that affects skeletal muscles and various systemic organs, such as the skin, lungs, heart, and joints (1, 2). Currently, myositis-specific autoantibodies (MSAs) and myositis-associated autoantibodies are widely used in clinical practice because they help not only in the diagnosis of IIM, but are also useful for the subcategorization and prediction of the clinical characteristics, disease course, and prognosis of the disease.

The anti-melanoma differentiation-associated gene 5 (MDA5) antibody, which is an MSA, was first reported to be an anti-clinically amyopathic dermatomyositis-140 (anti-CADM-140) antibody specific to CADM in a Japanese cohort in 2005, which showed a strong association with rapidly progressive interstitial lung disease (ILD) (3). The anti-MDA5 antibody can be identified in patients with classic dermatomyositis (DM); however, those with anti-MDA5 antibody (anti-MDA5 [+]) DM/CADM rarely present with critical muscle symptoms, making early detection challenging (4).

Previous studies have shown that 6–75% of the patients with polymyositis (PM)/DM have concomitant cardiac disorders (e.g., heart failure, coronary artery disease, conduction abnormalities, and abnormalities on electrocardiography [ECG] or ultrasound cardiography [UCG]) (5, 6). Nonetheless, there is a paucity of data regarding cardiac involvement in anti-MDA5 (+) DM/CADM (7). Therefore, in this study, we investigated whether cardiac involvement is associated with anti-MDA5 (+) DM/CADM. This is the first report on a specific cardiac abnormality observed in patients with anti-MDA5 (+) DM/CADM.

## Materials and Methods

### Patients and Clinical Data

Patients with PM/DM/CADM who were newly diagnosed and admitted to Kyoto University Hospital for remission induction therapy from March 2016 to September 2021 were enrolled in this study. The patients’ medical records were retrospectively reviewed and analyzed. The remission phase was defined as the period during which the steroid dose could be reduced for more than 3 months after treatment without a relapse. All the patients who underwent ECG and UCG were included. PM and DM were diagnosed according to the criteria outlined by Bohan and Peter (1, 2). CADM was diagnosed for the patients who had skin lesions typical of DM, but little or no muscle-related symptoms (8). ILD was diagnosed based on a physical examination, pulmonary function tests, chest radiography, high-resolution chest computed tomography, and the presence of respiratory symptoms. We collected the included patients’ laboratory data before treatment (just around the admission date). The patients’ serum was screened for MSAs using [35S]methionine-labeled protein/RNA immunoprecipitation and ELISA (MESACUP anti-MDA5, MESACUP anti-TIF1-γ, MESACUP anti-ARS, and MESACUP anti-Mi-2; MBL Co., Ltd., Nagoya, Japan), as previously described (4, 9, 10). We divided the patients into an anti-MDA5 (+) group and an anti-MDA5 (-) group and compared the clinical, laboratory, and physiological data between the two groups.

### Ethical Considerations

This study was conducted in accordance with the Declaration of Helsinki and approved by the ethics committee of Kyoto University Graduate School and Faculty of Medicine (approval number: E458). Written informed consent was obtained from all the patients prior to enrollment.

### Electrocardiography and Ultrasound Cardiography

ECG and UCG findings were reviewed at the first admission for remission induction. The typical ECG wave forms were analyzed using the QP-170D ECG Viewer software version 9 (Nihon Kohden, Tokyo, Japan). Two cardiologists (Y.K. and E.T.K.) reviewed the ECG and UCG findings. ECG and UCG parameters were then compared between the anti-MDA5 (+) and anti-MDA5 (-) groups.

### Statistical Analysis

The differences between the anti-MDA5 (+) and anti-MDA5 (-) groups were analyzed using an unpaired t-test for the continuous variables and a chi-square test for the categorical variables. Data are expressed as the mean ± standard deviation for the continuous variables and as percentages for the categorical variables, unless otherwise noted. All the statistical analyses were performed using the JMP Pro statistical package version 16.0.0 J (SAS Institute Inc., Cary, NC, USA). The differences were considered statistically significant at a two-tailed *p* < 0.05.

## Results

### Patient Characteristics

**Figure 1:**
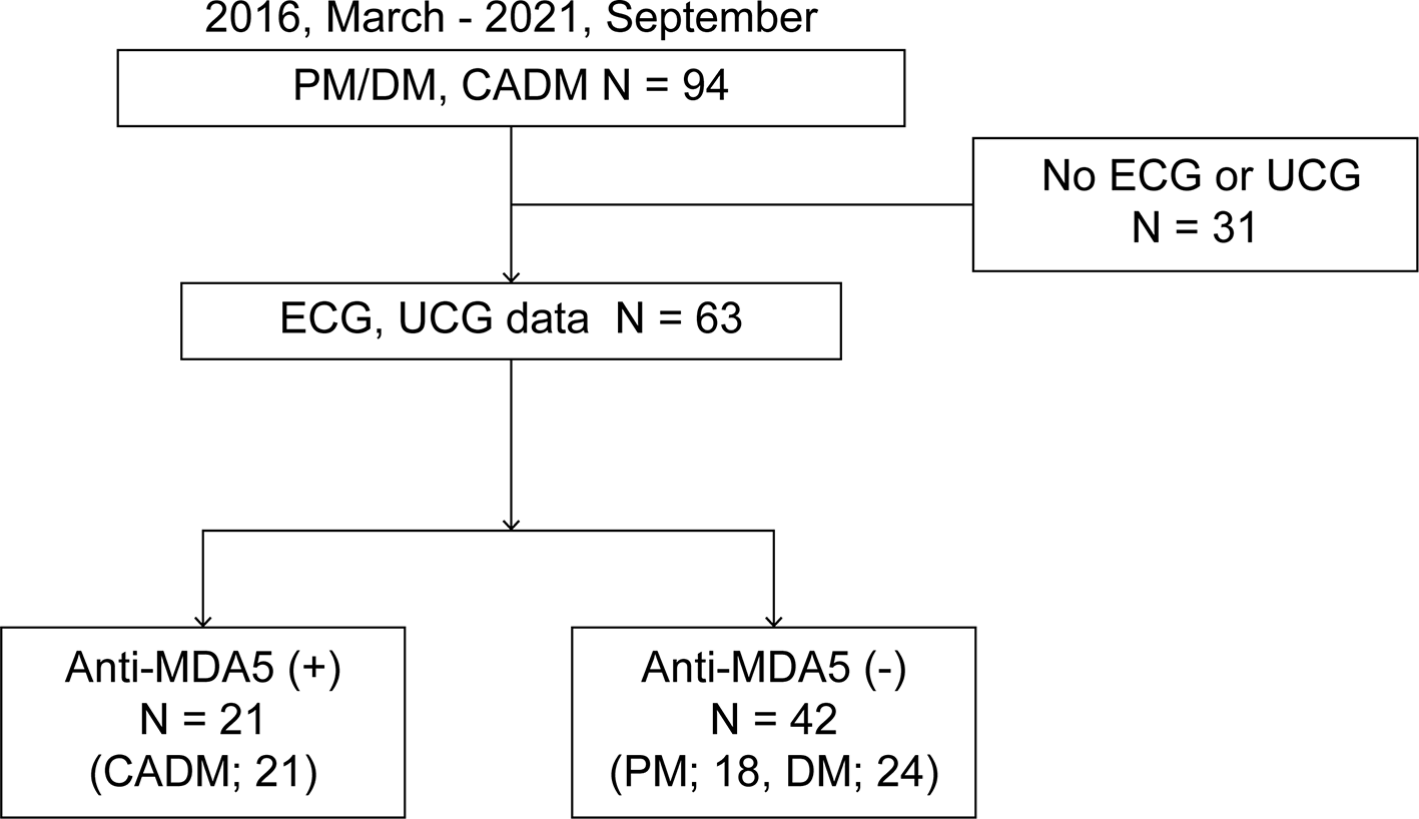
Study flowchart.

Of the 94 enrolled patients with PM/DM/CADM, 63 underwent both ECG and UCG on admission. We divided these patients into an anti-MDA5 (+) group (n = 21) and an anti-MDA5 (-) group (n = 42), the latter of which comprised fourteen anti-ARS (+), five anti-TIF1-γ (+), seven anti-SRP (+), two anti Mi-2(+), one anti Ku (+), one anti NXP2 (+) and twelve MSA-negative patients. All patients in the anti-MDA5 (+) group had CADM; conversely, all patients in the anti-MDA5 (-) group had PM/DM (**Figure 1**).

We compared the baseline clinical data between the anti-MDA5 (+) and anti-MDA5 (-) groups (**Table 1**). ILD and skin ulcers were observed significantly more frequently in the anti-MDA5 (+) group than in the anti-MDA5 (-) group. White blood cell, lymphocyte, serum creatine phosphokinase, lactate dehydrogenase, aldolase, K, and Ca levels were significantly lower in the anti-MDA5 (+) group than in the anti-MDA5 (-) group (*p* < 0.01). Platelet, AST, ALT, Na, and Cl levels were also significantly lower in the anti-MDA5 (+) group than in the anti-MDA5 (-) group (*p* < 0.05). On or after admission, four patients in the anti-MDA5 (+) group and six patients in the anti-MDA5 (-) group showed obvious cardiac abnormality (e.g., diastolic dysfunction, heart failure, myocarditis).

**Table 1:** Patient characteristics.

	MDA5 (+) (N = 21)	MDA5 (-) (N = 42)	p value
**Age (years)**	55.6 ± 9.6	58.9 ± 16.6	0.4
**Female (%)**	66.7	73.1	0.59
**Disease duration (Months)**	6.2 ± 12.4	4.8 ± 3.0	0.49
**ILD (%)**	100	45.2	<0.01
**Heart disease (%)**	14.3	12.5	0.8
**Malignancy (%)**	4.7	16.7	0.18
**Skin ulcer (%)**	33.3	2.4	<0.01
**WBC (/μL)**	5959.0 ± 2557.9	8553.1 ± 3625.9	<0.01
**Lymphocytes (/μL)**	730.0 ± 227.4	1368.6 ± 558.2	<0.01
**Neutrophils (/μL)**	4677.6 ± 2450.0	6193.1 ± 3394.5	0.08
**Platelets (10^4^/μL)**	23.2 ± 5.8	28.0 ± 8.4	0.03
**KL-6 (U/mL)**	775.0 ± 292.9	683.5 ± 860.0	0.65
**ESR (mm/1 h)**	69.7 ± 101.6	40.4 ± 35.9	0.13
**CPK (IU/L)**	227.7 ± 258.7	3043.3 ± 3724.3	<0.01
**LDH (IU/L)**	355.8 ± 105.5	617.0 ± 326.1	<0.01
**AST (IU/L)**	69.7 ± 65.4	123.2 ± 107.3	0.04
**ALT (IU/L)**	44.1 ± 30.7	83.0 ± 71.9	0.02
**CRP (mg/dL)**	0.7 ± 0.9	1.2 ± 1.9	0.33
**Ferritin (ng/mL)**	630.9 ± 664.3	352.7 ± 555.6	0.13
**Aldolase (U/L)**	7.2 ± 2.1	34.1 ± 29.4	<0.01
**Na (mEq/L)**	137.2 ± 3.9	139.2 ± 2.3	0.01
**K (mEq/L)**	3.8 ± 0.3	4.2 ± 0.3	<0.01
**Cl (mEq/L)**	101.4 ± 4.2	103.5 ± 2.2	0.01
**Ca (mg/dl)**	8.9 ± 0.3	9.2 ± 0.4	<0.01
**TP (g/dL)**	6.7 ± 0.4	6.9 ± 0.9	0.38
**ALB (g/dL)**	3.3 ± 0.5	3.4 ± 0.7	0.46

Statistical significance: p < 0.05.

Continuous variables were compared using the unpaired t-test, and are expressed as the mean ± standard deviation. Categorical variables were compared using the chi-square test, and are expressed as percentages (%), unless otherwise noted. Ca refers to corrected total serum calcium (protein-bound calcium, complexed calcium, and ionized calcium). The correction formula is as follows; Corrected Ca value (mg/dl) = serum total Ca value + 4- serum albumin value.

ILD, interstitial lung disease; WBC, white blood cell count; KL-6, Krebs von den Lungen-6; ESR, erythrocyte sedimentation rate; CPK, creatine phosphokinase; LDH, lactate dehydrogenase; AST, aspartate aminotransferase; ALT, alanine aminotransferase; CRP, C-reactive protein; TP, total protein; ALB, albumin.

### ECG and UCG Characteristics of Patients With Anti-MDA5 (+) CADM

Next, we analyzed the ECG findings for each group. While the electrolyte levels in all patients were within the normal range on admission, patients with anti-MDA5 (+) CADM tended to show low T waves during the active phase of the disease, but T wave restoration during the remission phase (**Supplementary Figure 1**). At the active phase, the most frequent comment regarding the ECG analysis in the anti-MDA5 (+) group was “non-specific T-wave abnormalities” (57%, 12/21); this is the” low T-wave in multiple leads”. The next most frequent comments were “normal range” (19%, 4/21) and “ST-T changes”, such as ST depression (19%, 4/21). In the anti-MDA5(-) group, comments included “normal range” (33%, 14/42), “non-specific T-wave abnormalities” (24%, 10/42), and “ST-T changes” (14%, 6/42). We further analyzed the various ECG parameters (including the R and T wave amplitudes, as well as other categories) and compared them between the groups (**Table 2**). There were no significant differences in the heart rate, QT interval, and R wave amplitudes between the anti-MDA5 (+) and anti-MDA5 (-) groups; however, the T wave amplitudes from multiple leads were significantly lower for the anti-MDA5 (+) group than for the anti-MDA5 (-) group (I, II, and V4–6, *p* < 0.01; aVF and V3, *p* < 0.05). The T wave amplitude from the aVR lead, which reflects the reverse direction of the myocardial electrical flow from the other leads, was higher for the anti-MDA5 (+) group than for the anti-MDA5 (-) group (*p* < 0.01). Among the MSA-positive patients (MDA5, ARS, TIF1-γ, SRP), the anti-MDA5 (+) group tended to show a lower T wave amplitude than the patients with ARS, TIF1-γ, and SRP antibody (data not shown).

**Table 2:** ECG parameters in the active phase.

ECG	HR (bpm)	QT Int (ms)				
MDA5 (+), n = 21	79.5 ± 13.1	369.9 ± 30.4				
MDA5 (-), n = 42	78.9 ± 14.7	374.1 ± 28.0				
*p*-value	0.43	0.97				
**R wave (μV)**	**Ⅰ**	**II**	**III**	**aVR**	**aVL**	**aVF**
MDA5 (+), n = 21	554.8 ± 266.0	757.1 ± 293.1	339.8 ± 313.1	61.4 ± 62.5	284.5 ± 241.7	522.1 ± 299.5
MDA5 (-), n = 42	464.9 ± 234.5	740.- ± 451.8	485.7 ± 444.1	82.6 ± 99.3	274.9 ± 269.9	594.2 ± 444.1
*p*-value	0.24	0.32	0.09	0.8	0.29	0.17
**T wave (μV)**						
MDA5 (+), n = 21	85.0 ± 43.0	113.1 ± 84.8	34.3 ± 72.4	-97.4 ± 57.6	27.9 ± 40.8	65.5 ± 78.9
MDA5 (-), n = 42	137.7 ± 63.1	185.8 ± 107.4	40.0 ± 111.8	-159.5 ± 71.0	47.6 ± 72.3	118.6 ± 102.5
*p*-value	<0.01	<0.01	0.6	<0.01	0.26	0.04
**T/R ratio**						
MDA5 (+), n = 21	0.21 ± 0.19	0.16 ± 0.16	0.09 ± 0.82	-1.98 ± 1.51	0.04 ± 0.65	0.12 ± 0.19
MDA5 (-), n = 42	0.45 ± 0.54	0.42 ± 0.46	0.32 ± 0.96	-2.70 ± 2.20	0.34 ± 0.92	0.33 ± 0.39
*p*-value	0.06	0.02	0.35	0.17	0.2	0.03
**R wave (μV)**	**V1**	**V2**	**V3**	**V4**	**V5**	**V6**
MDA5 (+), n = 21	168.6 ± 102.8	386.4 ± 212.8	596.2 ± 342.2	1206.0 ± 601.0	1627.9 ± 563.9	1286.2 ± 387.8
MDA5 (-), n = 42	221.5 ± 200.1	482.6 ± 375.9	725.0 ± 512.5	1222.7 ± 663.9	1342.4 ± 542.1	1093.7 ± 461.5
*p*-value	0.08	0.12	0.14	0.64	0.08	0.3
**T wave (μV)**						
MDA5 (+), n = 21	44.3 ± 116.4	286.7 ± 195.6	315.0 ± 206.7	238.6 ± 192.0	159.8 ± 145.7	114.3 ± 117.1
MDA5 (-), n = 42	27.9 ± 115.3	349.9 ± 222.1	452.1 ± 251.0	405.7 ± 226.6	316.4 ± 180.8	229.5 ± 137.9
*p*-value	0.6	0.28	0.04	<0.01	<0.01	<0.01
**T/R ratio**						
MDA5 (+), n = 21	0.30 ± 0.86	0.79 ± 0.64	0.69 ± 0.62	0.28 ± 0.34	0.12 ± 0.13	0.11 ± 0.11
MDA5 (-), n = 42	0.04 ± 1.49	0.96 ± 1.04	0.83 ± 0.69	0.42 ± 0.33	0.30 ± 0.34	0.23 ± 0.15
*p*-value	0.47	0.25	0.46	0.14	0.02	<0.01

Statistical significance: p < 0.05.

The amplitudes of the R and T waves on ECG were calculated for all the leads.

HR, heart rate; QT int, QT interval.

According to the Minnesota code classification system, a low T wave is defined as a T wave amplitude/R wave amplitude (T/R ratio) <0.1 (11, 12). The T/R ratios for the anti-MDA5 (+) group were lower for some of the leads (V6 *p* < 0.01; II, aVF and V5 *p* < 0.05). The four anti-MDA 5 (+) patients with obvious cardiac abnormality had six or more leads with low T-wave.

Because ILD was found to have been present as a complication in the anti-MDA5 (+) CADM group at a significantly higher frequency (100%) than in the anti-MDA5 (-) group (45.2%), we conducted an additional analysis to examine whether the incidence of ILD affected the ECG findings. First, we compared ECG parameters between the anti-MDA5 (-) patients with and without ILD (n = 22 and 20, respectively). The non ILD group had significantly higher T waves in the III lead, and conversely, significantly lower T waves in the aVL lead. We could not conclude that the presence of ILD was related to the number of lead with low T wave (**Supplementary Table 1**). Moreover, there were no significant differences in the ECG characteristics between the MDA5 (-) non-ILD group and the acute or subacute ILD (A/S-ILD) groups (n = 22 and 13, respectively; **Supplementary Table 2**). Subsequently, ECG parameters were compared between patients in the anti-MDA5 (+) group (n = 21) and those in the anti-MDA5 (-) group with ILD. The anti-MDA5 (+) group showed a significantly lower T wave than the anti-MDA5 (-) group with ILD (**Supplementary Table 3**). Furthermore, when the anti-MDA5 (-) A/S-ILD group was set as the control group and compared with the anti-MDA5 (+) A/S-ILD group (n=18), the anti-MDA5 (+) A/S-ILD group still showed a significantly lower T wave (**Supplementary Table 4**).

Collectively, the low T wave amplitude recorded on admission was a characteristic feature of the anti-MDA5 (+) group, independent of the presence of ILD.

Next, we analyzed the UCG data (**Table 3**). There appeared to be no difference in the contractile function (left ventricular end-diastolic diameter, left ventricular end-systolic diameter, and left ventricular ejection fraction) between the anti-MDA5 (+) and anti-MDA5 (-) groups. With respect to cardiac diastolic function, the E wave and A wave of the transmitral flow velocity pattern, and movement speed of the mitral valve annulus at the septum (Sep e’) were significantly lower (*p* < 0.05) for the anti-MDA5 (+) group than for the anti-MDA5 (-) group. However, the E/e’ ratio was not significantly different between the groups, and no significant differences in the diastolic interventricular septum or left ventricular posterior wall thicknesses were observed; in almost all cases of the anti-MDA5 (+) group, there was normal cardiac function on echo. These results suggest that subclinical damage of the left ventricle exists in patients with anti-MDA5 (+) CADM (13). In this study, two patients (10%) had diastolic dysfunction or diastolic failure in the anti-MDA5 (+) group and three (7%) in the anti-MDA5 (-) group.

**Table 3:** UCG parameters in the active phase.

UCG	MDA5 (+) n = 21	MDA5 (-) n = 42	p value
**LVEF (%)**	67.7 ± 9.5	69.7 ± 6.0	0.31
**LVDd (mm)**	41.5 ± 4.6	42.4 ± 4.8	0.44
**LVDs (mm)**	25.9 ± 4.7	25.9 ± 4.1	0.99
**IVSTd (mm)**	9.3 ± 1.6	9.7 ± 2.7	0.52
**PWTd (mm)**	9.4 ± 1.7	9.5 ± 1.8	0.94
**LADs (mm)**	33.8 ± 5.6	32.7 ± 4.2	0.87
**E wave (m/sec)**	0.58 ± 0.14	0.74 ± 0.23	<0.01
**A wave (m/sec)**	0.61 ± 0.19	0.74 ± 0.18	0.01
**E/A**	1.1 ± 0.61	1.1 ± 0.51	0.94
**DcT (msec)**	218.3 ± 49.4	238.5 ± 73.5	0.27
**Sep E/e’**	9.5 ± 4.3	9.5 ± 3.7	0.8
**Sep e’ (cm/sec)**	6.7 ± 2.0	8.9 ± 3.9	0.02
**Sep a’ (cm/sec)**	10.3 ± 3.4	10.1 ± 2.1	0.75

Statistical significance: p < 0.05.

LVEF, left ventricular ejection fraction; LVDd, left ventricular diameter at end-diastole; LVDs, left ventricular internal dimension in systole; IVSTd, interventricular septal thickness at end-diastole; PWTd, posterior LV wall thickness at end-diastole; LADs, left atrial dimension in systole; E wave, early diastolic filling velocity; A wave, atrial filling velocity; E/A, E wave/A wave ratio; DcT, deceleration time; Sep E/e’ ratio, E wave/e’ ratio at the septum of the left ventricle; Sep e’, peak early diastolic mitral annular velocity at the septum of the left ventricle; Sep a’, peak atrial systolic mitral annular velocity at the septum of the left ventricle.

### Recovery of Low T Waves in Anti-MDA5 (+) CADM After Treatment

For the anti-MDA5 (+) group, we investigated whether ECG changes would be reversible after immunosuppressive therapy. During the active phase of the disease, the number of leads in the 12-lead ECG (not including the aVR lead) that showed a low T wave was higher for the anti-MDA5 (+) group than for the anti-MDA5 (-) group (*p* < 0.01; **Table 4**) upon admission. However, during the remission phase, there was no significant difference in the number of leads with low T waves between the two groups. In summary, the low T wave can be reversible, and this is associated with disease activity in the anti-MDA5 (+) group. Four anti-MDA5 (+) patients who had heart dysfunction improved clinically and still had low T waves even though the number of leads with low T wave decreased. At the remission phase, due to small sample size, there was no significant difference between the groups, but the E wave, A wave, and Sep e’ in the anti-MDA5 (+) group were still lower (**Supplementary Table 5**).

**Table 4:** Number of leads with a low T wave in the active and remission phases.

		Number of Leads		*p*-value
		0–1	>1	
**Active phase**	**MDA5 (+)**	2	12	<0.01
	**MDA5 (-)**	14	7	
**Remission phase**	**MDA5 (+)**	7	7	n.s.
	**MDA5 (-)**	9	12	

Statistical significance: p < 0.05; n.s., not significant.

“Low T wave” is defined according to the major ECG criteria (Minnesota code) as a T/R ratio <0.1. The T/R ratio at the aVR lead was excluded from the analysis. We counted the number of leads with Low T wave at 11 leads except for aVR (V1-6, I, II, III, aVL, aVF leads) for each patient. ‘>1’ means that there are more than two leads with a low T wave; ‘0-1’ means that there is less than one. The p-value reflects the results of the analysis between the anti-MDA5 (+) and anti-MDA5 (-) groups.

### Relationship Between the Number of Low T Waves, Clinical Data, and Echocardiography

Next, we performed a sub-analysis to see if the number of T waves was related to clinical and echocardiographic data within the anti-MDA5 (+) group. Regarding ECG findings, we divided patients into three groups, according to the number of leads with low T wave: Group A, 0–2 leads (N = 7); Group B, 3–5 leads (N = 6); and Group C, 6 or more leads (N = 8). Group C showed lower T wave amplitudes at multiple leads (**Supplementary Figure 2**). The results showed no significant difference in the frequency of skin ulcers. Epidemiology at admission showed that the percentage of female patients was 100% (N = 7) in Group A, 83% (N = 5) in Group B, and 25% (N = 2) in Group C. All the four patients who had obvious cardiac abnormality were in Group C and were male. Blood counts showed that the number of WBC and neutrophils, but not lymphocytes and platelets, tended to decrease in Group B and C (**Supplementary Figure 3**). Blood biochemical tests at admission showed that Group B and C tended to have higher CPK and CRP levels; there seemed to be no difference between the three groups in ferritin and KL-6 levels (**Supplementary Figure 4**). Regarding clinical data and UCG, due to the small sample size, there was no significant difference between the patients that, based on ECG findings, did not completely recover and those that fully recovered (data not shown); the group that did not recover included five men from Group C, including the four patients who had obvious cardiac abnormality.

Collectively, patients with low T wave at multiple leads may develop heart dysfunction or heart failure in anti-MDA5 (+) CADM. The number of leads with low T wave may be associated with some clinical data (e.g., number of WBCs, neutrophils, or levels of CPK and CRP). Even if anti-MDA5 (+) patients achieve remission, some may still have abnormalities on ECG and UCG parameters and cardiac damage.

## Discussion

To the best of our knowledge, this is the first report on cardiac involvement in an anti-MDA5 (+) CADM cohort. In this study, we found characteristic subclinical ECG and UCG abnormalities in patients with anti-MDA5 antibodies (a reversible low T wave on ECG and a low E wave, A wave and Sep e’ on UCG), suggesting the extent of cardiac damage. Although there were lower levels of electrolytes in the anti-MDA5 (+) group, there were no severe electrolyte abnormalities that could account for the abnormal ECG findings. With respect to the pathophysiology of anti-MDA5 (+) DM/CADM, elevated levels of several cytokines and chemokines, including interleukin (IL)-6, IL-8, IL-10, IP-10, sCD163, YKL-40, and IFN-α, have been reported (14–17). Several inflammatory cytokines, such as TNFα, the IL-1 family, IL-6, IL-8, IL-10, IL-18, and IFNα have also been shown to play a pathological role in various heart diseases (18). IL-8 stimulates the expression of Na-K^+^ or Ca^+^ channels and affects the flow of Ca^+^ or K^+^ (19). IL-10, an anti-inflammatory cytokine, correlates with heart inflammation or dysfunction in cardiomyopathy, acute myocarditis, and Takotsubo cardiomyopathy (20–22). Daily IL-18 administration has been reported to induce myocardial dysfunction in BALB/c mice (23), and interferon therapy can lead to reversible cardiomyopathy (24, 25). Thus, a prominent elevated levels of several inflammatory cytokines can cause myocardial impairment in patients with anti-MDA5 (+) DM/CADM.

One hypothesis is that myocardial damage is due to pathological autoantibodies. There have been previous reports of pathological autoantibodies as components of heart damage in mouse models (e.g., anti-myosin antibody, anti-cardiac troponin I antibody) (26). However, it is unclear why anti-MDA5 (+) CADM would have such an abnormal ECG and echocardiography finding. Since the T-wave finding is abnormal, one possibility is that pathological autoantibodies are being produced that causes a defect in ion channels involved in repolarization.

We also speculate that viral infection may be a trigger, with viral-induced myocarditis leading to the development of anti-MDA5 (+) CADM. It has long been suspected that viruses may be involved in the development of PM/DM (e.g., Parvo virus, Coxsackie virus) (27–29). The type1 IFN pathway, which has antiviral effects, is activated in PM/DM (30). There are some reports that seasonality and regionality are related to the development of anti-MDA5 (+) CADM (31, 32). As the phenotype of CADM is different in Asia, Europe, and the United States, the causative virus may be different in each region (33). Thus, environmental factors such as viruses may contribute to the development of the disease. Consistent with this idea, an IFN signature is activated in anti-MDA5 (+) CADM (34).

MDA5 is a viral sensor in the cell, which plays an important role in detecting picornaviruses (e.g., Coxsackie virus) that cause myocarditis and encephalitis (35, 36). During the preparation of this report, COVID-19 became widespread. Recently, there have been increasing reports of an association between COVID-19 infection and autoimmune diseases. COVID-19 infection has been reported to cause the production of autoantibodies and promote the development of autoimmune diseases (37, 38). In COVID-19 infection, MDA5 also acts as a sensor (39), and there has been a report of anti-MDA5 antibody positivity after infection (38). Furthermore, it has been reported that the clinical symptoms, CT images and blood test findings (e.g., hypokalemia, and high levels of ferritin, CPK and CRP) of COVID-19 pneumonia are similar to those of anti-MDA5 (+) CADM (40, 41). COVID-19 infection also results in a high frequency of abnormalities on cardiac MRI and echocardiography, even when the patient is asymptomatic (42, 43). In summary, COVID-19-induced pneumonia and associated autoimmunity provides potential insight into the pathogenesis of anti-MDA5 antibody positive CADM. Since anti-MDA5 (+) CADM was recognized before the spread of COVID-19, we assume that some viruses (recognized by MDA5), which are common in Asia, may be risk factors for the development of anti-MDA5 (+) CADM, including myocardial damage.

We observed that the low T waves recorded in the anti-MDA5 (+) group during the active disease phase were reversible after treatment (**Supplementary Figure 1** and **Table 4**). However, some patients with multiple low T waves developed obvious heart dysfunction and the abnormal T wave did not fully resolve. This suggests that aberrant immune responses associated with anti-MDA5 (+) DM/CADM disease activity affect changes in electrical activity of the heart, leaving some patients with cardiac damage even in remission. We performed a sub-analysis to determine if there was any association between the number of low T waves and clinical data. Although our sample size was small, WBC, neutrophil, CPK and CRP seemed to be associated with the number of leads with low T wave; this may reflect structural damage to the heart. Interestingly, patients with heart dysfunction (e.g., heart failure, myocarditis, and diastolic dysfunction) who had more leads with low T waves were also more likely to be male. The one reported case of anti-MDA5 (+) cardiomyopathy was a 55-year-old man (7). In a model of coxsackie virus myocarditis, males have been reported to be more severely affected (44). In anti-MDA5 (+) DM, muscle, skin, and joint symptoms have been reported to be related to sex (33). One phenotype with high CPK levels and muscle symptoms seemed to be common in male patients (45). Our data suggest that men with anti-MDA5 (+) CADM may be more prone to severe myocardial damage and present low T wave in multiple leads. However, in the patients enrolled, smoking history, hypertension, hyperlipidemia, and diabetes, which are risk factors for heart disease, seemed to be more prevalent in men (data not shown). We would like to collect more cases in the future to clarify the relationship between ECG findings, cardiac function, and clinical data. Investigating the mechanism underlying this electrical abnormality may lead to an understanding of the pathophysiology of anti-MDA5 (+) DM/CADM, which often shows amyopathic or hypomyopathic phenotypes.

In our study, cardiac function was nearly normal for patients with anti-MDA5 antibodies. Some of the UCG parameters (namely, the E wave, A wave and Sep e’) showed significant differences between the groups. In the remission phase, the values of E wave, A wave, and Sep e were not significantly different, likely due to the small sample size; however, as in the active phase, they remained lower in the anti-MDA5 (+) group than in the anti-MDA5 (-) group, suggesting that some cardiac damage may remain even after remission. The E wave represents the early diastolic filling velocity, which is one of the transmitral flow velocity patterns. When the left ventricular diastolic function decreases, left ventricular relaxation is delayed, and the E wave also decreases. Sep e’ is the peak early diastolic mitral annular velocity at the septum (46). A low Sep e’ is an indicator of diastolic disturbance. Previous studies have reported that PM/DM patients are sometimes complicated with left ventricular diastolic dysfunction (LVDD) (12–42%) (6, 47–50). During diastolic dysfunction, the A wave generally tends to be high; therefore, we could not conclude why the A wave was lower in the anti-MDA5 (+) group. The low T-wave and echocardiographic findings suggest that there is an unknown cardiac pathology at this time that does not fit the definition of diastolic dysfunction. In the future, we will try to clarify the cause. Although our findings did not meet the UCG diagnostic criteria for diastolic disturbance (13), our data suggest the possible presence of cardiac involvement in patients with anti-MDA5 antibodies. In our facility, two CADM patients with anti-MDA5 antibodies (10%) and three PM/DM patients (7%) and were diagnosed with LVDD. Although two of the CADM patients with anti-MDA5 antibodies died of respiratory failure, we speculate they may have ultimately had severe heart damage, such as diastolic failure.

We assumed that the ECG results for the anti-MDA5 (+) group were affected by the 100% ILD complication rate in this group; therefore, we conducted a sub-analysis to confirm our hypothesis. Even though ILD was present more frequently in the anti-MDA5 (+) group (**Table 1**), the ECG findings did not show an association with this observation (**Supplementary Tables 1**–**4**). Few studies have reported abnormal ECG findings in association with PM/DM with LVDD (51). In that sense, the ECG findings for the anti-MDA5-antibody positive patients are unique. ECG records cardiac muscle activity, and the T wave represents the period during which the ventricles transition from systole to diastole. A low T wave may be indicative of an electrical activity alteration and structural change. Further serial functional imaging tests may be useful for identifying the extent of cardiac involvement and understanding the clinical implications of these ECG changes. In the present study, most of the anti-MDA5-antibody positive cases had A/S-ILD, and there were three cases of no A/S-ILD (organizing pneumonia, ILD with chronic course), one of which had multiple leads with low T wave. In our department, we previously certified another six cases of anti-MDA5 (+) non-A/S-ILD, in which a few cases showed the same ECG findings. More cases need to be collected to determine if the ECG findings are also detectable in anti-MDA5 (+) CADM with non-A/S-ILD.

Our study has several limitations. First, this was a retrospective study; therefore, we lacked some clinical data for the included patients. A prospective study is needed to confirm our findings. Second, the clinical implications of a low T wave on ECG, and a low E wave, A wave and Sep e’ on UCG, require further assessment. However, our results may provide new insight into whether anti-MDA5 (+) DM/CADM is a multiorgan autoimmune disease. Therefore, further basic investigations are required.

In conclusion, patients with anti-MDA5 antibodies often have specific ECG and UCG findings that indicate subclinical heart involvement. As patients with anti-MDA5 antibodies have a poor prognosis if treatment is delayed, ECG may aid in early detection. Therefore, cardiac function screening and follow-ups for this cohort will be important, even if patients show only slight myocarditis or heart failure.

## Data Availability Statement

The original contributions presented in the study are included in the article/**Supplementary Material**. Further inquiries can be directed to the corresponding author at ranran@kuhp.kyoto-u.ac.jp.

## Ethics Statement

The studies involving human participants were reviewed and approved by the ethics committee of Kyoto University Graduate School and Faculty of Medicine (approval number: E458). The patients/participants provided their written informed consent to participate in this study.

## Author Contributions

TaM, YK, and RN designed the study. TaM and TS collected the data. TaM, YK, and EK reviewed the ECG and UCG data. TaM and YK analyzed the data. TaM, YK, EK, and RN interpreted the data. TaM and RN wrote the manuscript. All the authors reviewed and approved the manuscript.

## Funding

This work was supported by a Grant-in-Aid for Scientific Research from the Japan Society for the Promotion of Science (18K16145 for RN). The funding body had no role in the study design, the collection, analysis, and interpretation of the data, or in the writing of the manuscript.

## Conflict of Interest

RN has received research grants from Takeda and Medical & Biological Laboratories Co., Ltd., as well as speaker fees outside of the submitted work from Bristol Myers Squibb, Astellas Pharma Inc., Boehringer Ingelheim, Actelion Pharmaceuticals, and Mitsubishi Tanabe Pharma. HY has received a speaker fee from Boehringer Ingelheim. MT has received research grants and speaker fees from AbbVie GK, Asahi Kasei Pharma Corp., Astellas Pharma Inc., Ayumi Pharmaceutical Corp., Bristol Myers Squibb, Chugai Pharmaceutical Co., Ltd., Eisai Co., Ltd., Eli Lilly Japan K.K., Pfizer Inc., UCB Japan Co., Ltd., Janssen Pharmaceutical K.K., Mitsubishi Tanabe Pharma Corp., Novartis Pharma K.K., and Taisho Pharma Co., Ltd.

The remaining authors declare that the research was conducted in the absence of any commercial or financial relationships that could be construed as a potential conflict of interest.

## Publisher’s Note

All claims expressed in this article are solely those of the authors and do not necessarily represent those of their affiliated organizations, or those of the publisher, the editors and the reviewers. Any product that may be evaluated in this article, or claim that may be made by its manufacturer, is not guaranteed or endorsed by the publisher.
