# Supplementary material for: ECG Changes Through Immunosuppressive Therapy Indicate Cardiac Abnormality in Anti-MDA5 Antibody-Positive Clinically Amyopathic Dermatomyositis
Source: Front Immunol. 2022 Jan 7;12:765140. doi: 10.3389/fimmu.2021.765140 (PMC8776991; doi:10.3389/fimmu.2021.765140)
Supplement: Supplementary file 1 [file DataSheet_1.docx]

Supplementary Materials

# Supplementary Figures

#


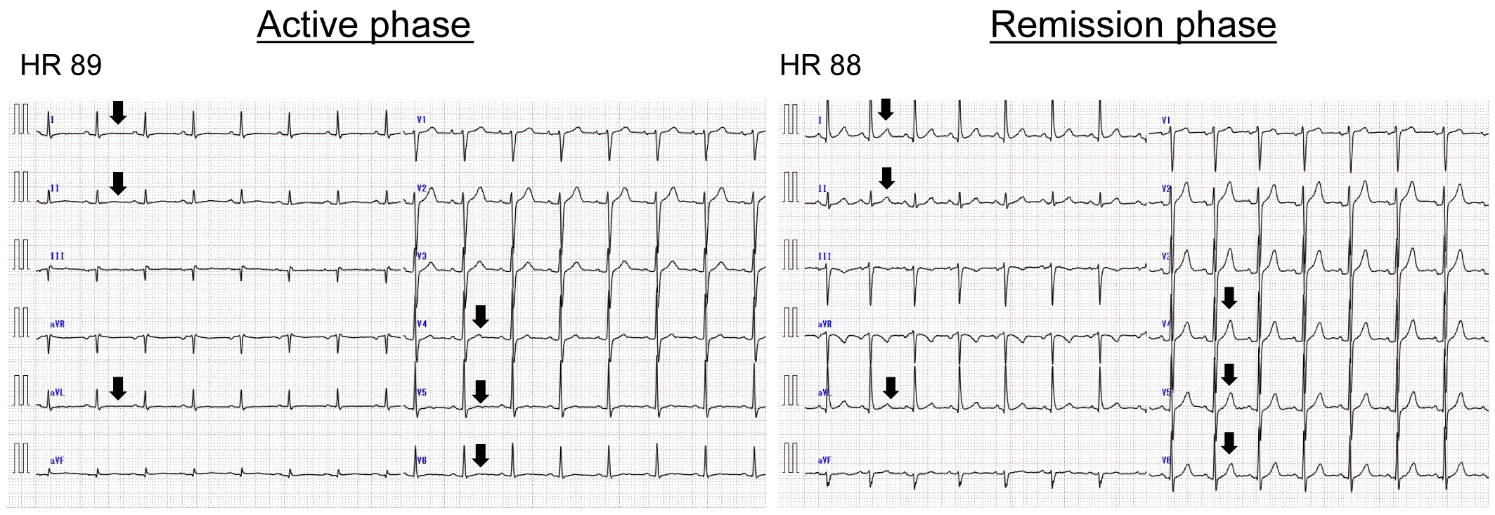


**Supplementary Figure 1.** ECG images of a patient with anti-MDA5 (+) CADM in the active and remission phases. Analysis of ECG findings revealed that low T waves recorded for the anti-MDA5 (+) group during the active disease phase were reversible after treatment.

**Supplementary Figure 2.** Amplitude of ECG in the anti-MDA5 (+) group (Ⅰ,Ⅱ, V4-6 leads). The patients with anti-MDA5 antibodies were divided into three groups, according to the number of leads with low T wave: Group A, 0–2 leads (N = 7); Group B, 3–5 leads (N = 6); and Group C, 6 or more leads (N = 8). Group C showed lower T wave amplitudes at multiple leads


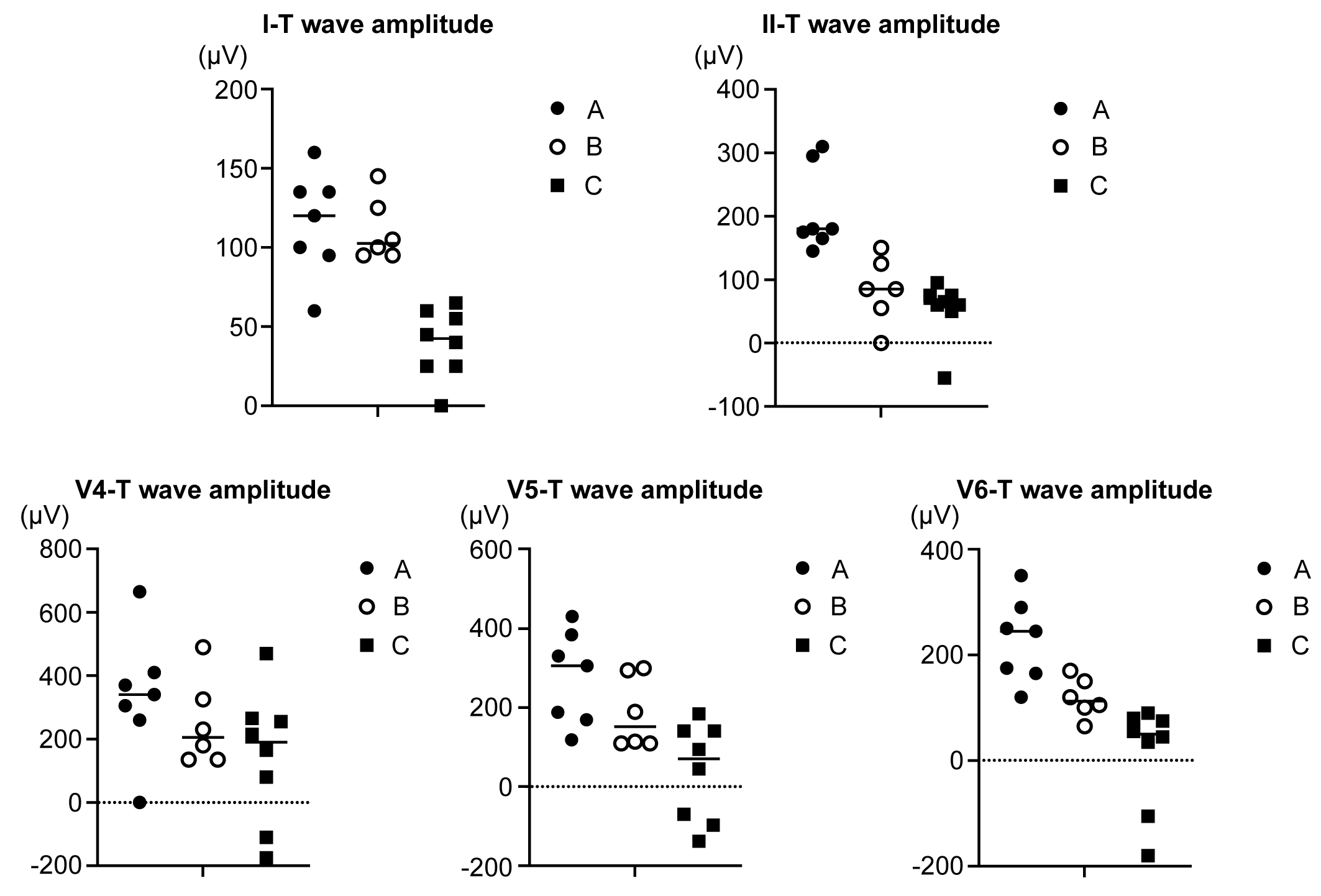


**Supplementary Figure 3.** Blood cell counts in the anti-MDA5 (+) group (WBC, Lymphocyte, Neutrophil, Platelet). The patients with anti-MDA5 antibodies were divided into three groups, according to the number of leads with low T wave: Group A, 0–2 leads (N = 7); Group B, 3–5 leads (N = 6); and Group C, 6 or more leads (N = 8). Group B and C showed lower number of WBC and Neutrophils, but not lymphocytes and platelets.


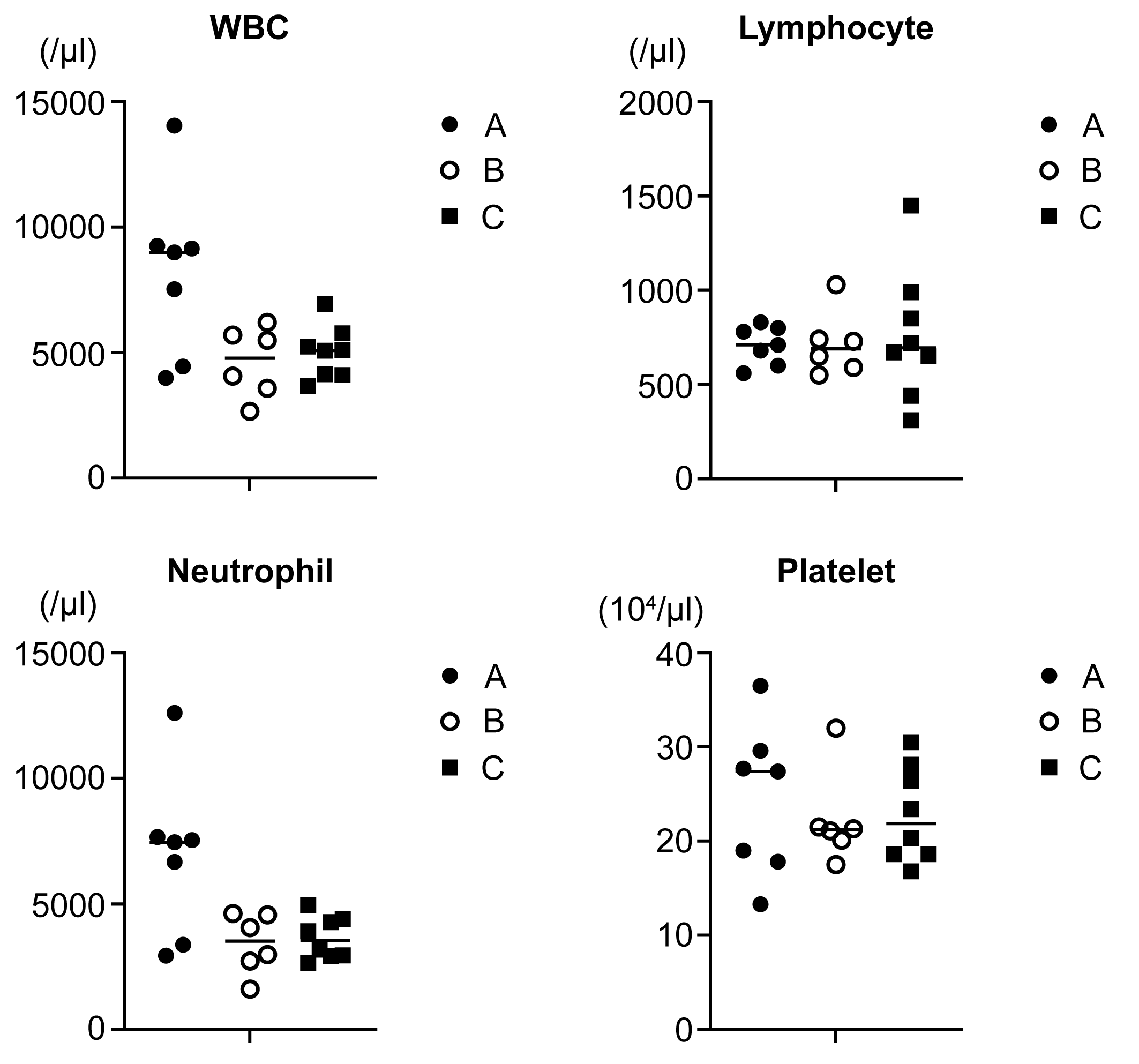


**Supplementary Figure 4.** Biochemical tests in the anti-MDA5 (+) group (CPK, CRP, KL-6, Ferritin). The patients with anti-MDA5 antibodies were divided into three groups, according to the number of leads with low T wave: Group A, 0–2 leads (N = 7); Group B, 3–5 leads (N = 6); and Group C, 6 or more leads (N = 8). Group B and C tended to have higher CPK and CRP levels.


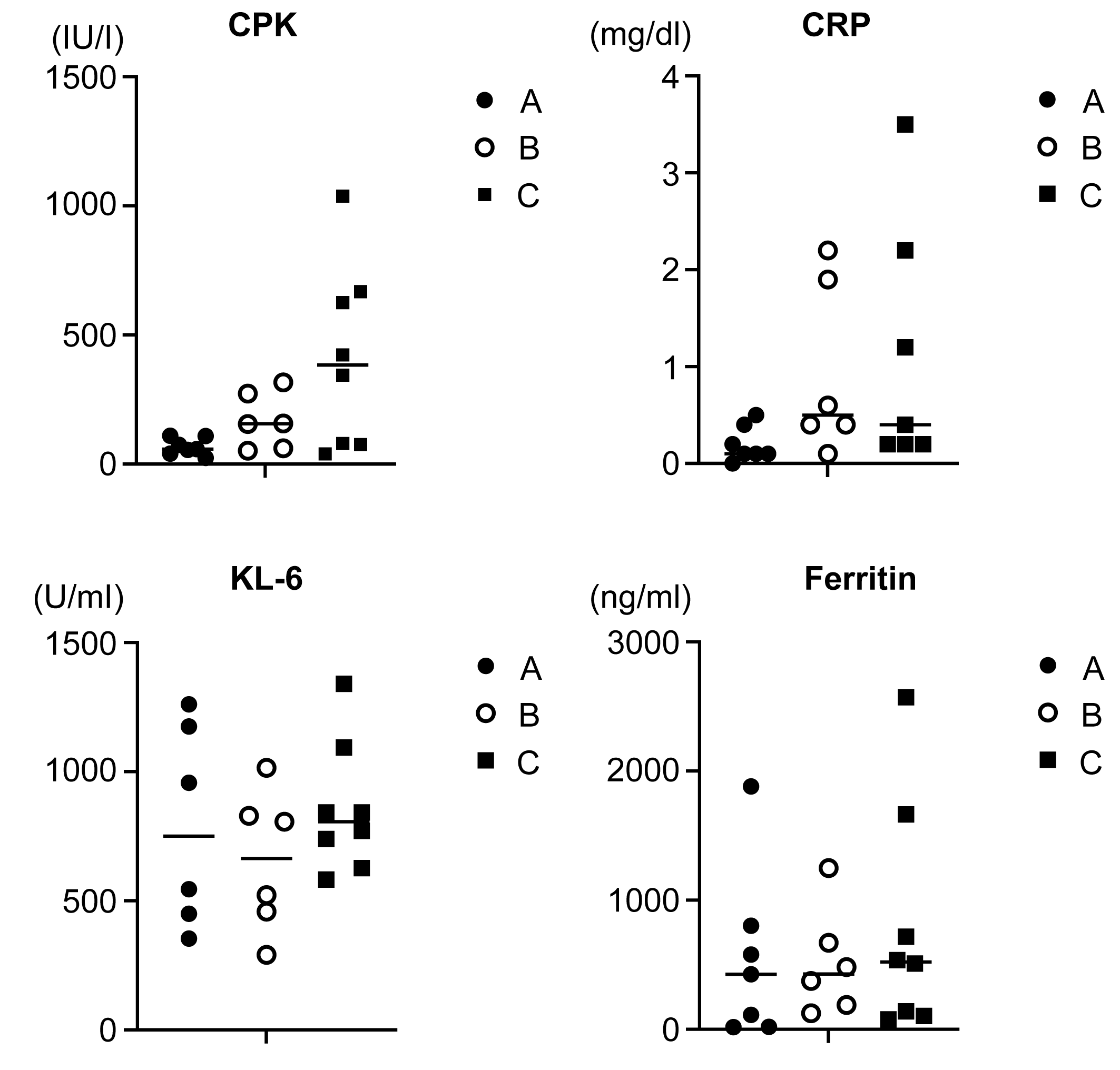


# Supplementary Tables

# Supplementary Table 1. Comparison of ECG parameters between the anti-MDA5 (-) patients with and without ILD in the active phase.

| **ECG** | **HR　(bpm)** | **QT Int (ms)** |  |  |  |  |
| --- | --- | --- | --- | --- | --- | --- |
| MDA5 (-) non ILD n = 22 | 77.1 ± 15.0 | 378.4 ± 32.5 |  |  |  |  |
| MDA5 (-) ILD n = 20 | 80.8 ± 14.0 | 369.4 ± 21.1 |  |  |  |  |
| p value | 0.44 | 0.31 |  |  |  |  |
|  |  |  |  |  |  |  |
| **R wave (μV)** | **Ⅰ** | **Ⅱ** | **Ⅲ** | **aVR** | **aVL** | **aVF** |
| MDA5 (-) non ILD n = 22 | 441.1 ± 145.0 | 661.4 ± 458.2 | 396.1±398.6 | 109.1 ± 114.4 | 256.6 ± 242.2 | 507.0 ± 431.1 |
| MDA5 (-) ILD n =20 | 491.0 ± 301.8 | 826.5 ± 428.3 | 584.3 ± 469.9 | 53.5 ± 68.4 | 295.0 ± 296.1 | 690.0 ± 438.4 |
| p value | 0.50 | 0.25 | 0.18 | 0.07 | 0.6 | 0.19 |
| **T wave (μV)** |  |  |  |  |  |  |
| MDA5 (-) non ILD n = 22 | 151.1 ± 56.1 | 166.6±.104.0 | 12.5 ± 111.3 | －155.5 ± 65.7 | 72.0 ± 67.5 | 90.0 ± 102.5 |
| MDA5 (-) ILD n = 20 | 123.0 ± 66.9 | 207.0±107.1 | 89.3 ± 101.8 | －164.0 ± 76.1 | 20.8 ± 67.8 | 150.0 ± 92.9 |
| p value | 0.16 | 0.23 | 0.03 | 0.71 | 0.02 | 0.06 |
| **T/R ratio** |  |  |  |  |  |  |
| MDA5 (-) non ILD n = 22 | 0.40 ± 0.25 | 0.49 ± 0.59 | 0.34 ± 1.20 | －1.93 ± 1.72 | 0.62 ± 1.01 | 0.32 ± 0.47 |
| MDA5 (-) ILD n = 20 | 0.50 ± 0.73 | 0.33 ± 0.24 | 0.31 ± 0.62 | －3.72 ± 2.36 | 0.03 ± 0.69 | 0.33 ± 0.28 |
| p value | 0.57 | 0.29 | 0.92 | 0.92 | 0.05 | 0.9 |
|  |  |  |  |  |  |  |
| **R wave (μV)** | **V1** | **V2** | **V3** | **V4** | **V5** | **V6** |
| MDA5 (-) non ILD n = 22 | 239.3 ± 228.3 | 494.3 ± 417.4 | 722.7 ± 518.7 | 1133.9 ± 578.7 | 1186.6 ± 539.3 | 994.8 ± 400.2 |
| MDA5 (-) ILD n = 20 | 202.0 ± 161.2 | 469.8 ± 323.7 | 727.5 ± 505.6 | 1320.5 ± 734.1 | 1513.8 ± 491.1 | 1202.5 ± 498.5 |
| p value | 0.56 | 0.84 | 0.98 | 0.38 | 0.05 | 0.15 |
| **T wave (μV)** |  |  |  |  |  |  |
| MDA5 (-) non ILD n = 22 | 2.0 ± 106.3 | 344.1 ± 201.5 | 427.5 ± 251.1 | 385.2 ± 223.6 | 311.6 ± 171.1 | 226.1 ± 124.0 |
| MDA5 (-) ILD n = 20 | 56.3 ± 118.2 | 356.3 ± 242.5 | 479.3 ± 248.0 | 428.2 ± 227.7 | 321.8 ± 190.8 | 233.3 ± 151.7 |
| p value | 0.13 | 0.86 | 0.52 | 0.55 | 0.86 | 0.87 |
| **T/R ratio** |  |  |  |  |  |  |
| MDA5 (-) non ILD n = 22 | -0.15 ± 0.67 | 0.68 ± 0.40 | 0.79 ± 0.74 | 0.35 ± 0.22 | 0.36 ± 0.45 | 0.26 ± 0.17 |
| MDA5 (-) ILD n = 20 | 0.24 ± 2.00 | 1.23 ± 1.36 | 0.87 ± 0.64 | 0.49 ± 0.40 | 0.24 ± 0.15 | 0.22 ± 0.13 |
| p value | 0.42 | 0.10 | 0.71 | 0.20 | 0.29 | 0.44 |

# Statistical significance: p < 0.05; Interstitial lung disease, ILD

**Supplementary Table 2.** Comparison of the ECG parameters between the anti-MDA5 (-) non ILD and anti-MDA5 (-) A/S-ILD groups in the active phase

| ECG | **HR　(bpm)** | **QT Int (ms)** |  |  |  |  |
| --- | --- | --- | --- | --- | --- | --- |
| MDA5 (-) non ILD, n = 22 | 77.1 ± 15.0 | 378.4 ± 32.5 |  |  |  |  |
| MDA5 (-) A/S-ILD, n = 13 | 78.7 ± 13.8 | 371.4 ± 16.9 |  |  |  |  |
| p value | 0.77 | 0.49 |  |  |  |  |
|  |  |  |  |  |  |  |
| **R wave (μV)** | **Ⅰ** | **Ⅱ** | **Ⅲ** | **aVR** | **aVL** | **aVF** |
| MDA5 (-) non ILD, n = 22 | 441.1 ± 145.0 | 661.4 ± 458.2 | 396.1 ± 398.6 | 109.1 ± 114.4 | 256.6 ± 242.2 | 507.0 ± 431.1 |
| MDA5 (-) A/S-ILD, n = 13 | 452.3 ± 261.0 | 831.9 ± 435.7 | 661.2 ± 500.9 | 53.5 ± 63.2 | 298.1 ± 322.6 | 748.5 ± 453.3 |
| p value | 0.88 | 0.30 | 0.10 | 0.13 | 0.68 | 0.14 |
| **T wave (μV)** |  |  |  |  |  |  |
| MDA5 (-) non ILD, n = 22 | 151.1 ± 56.1 | 166.6 ± 104.0 | 12.5 ± 111.3 | －155.5 ± 65.7 | 72.0 ± 67.5 | 90.0 ± 102.5 |
| MDA5 (-) A/S-ILD, n = 13 | 137.3 ± 77.8 | 224.2 ± 109.9 | 87.7 ± 114.6 | －181.5 ± 78.8 | 30.8 ± 77.5 | 157.3 ± 100.9 |
| p value | 0.56 | 0.14 | 0.07 | 0.31 | 0.12 | 0.08 |
| **T/R ratio** |  |  |  |  |  |  |
| MDA5 (-) non ILD, n = 22 | 0.40 ± 0.25 | 0.49 ± 0.59 | 0.34 ± 1.20 | －1.93 ± 1.72 | 0.62 ± 1.01 | 0.32 ± 0.47 |
| MDA5 (-) A/S-ILD, n = 13 | 0.53 ± 0.80 | 0.35 ± 0.25 | 0.34 ± 0.75 | －3.80 ± 2.21 | 0.09 ± 0.78 | 0.33 ± 0.30 |
| p value | 0.50 | 0.44 | 0.99 | 0.03 | 0.14 | 0.96 |
|  |  |  |  |  |  |  |
| **R wave (μV)** | **V1** | **V2** | **V3** | **V4** | **V5** | **V6** |
| MDA5 (-) non ILD, n = 22 | 239.3 ± 228.3 | 494.3 ± 417.4 | 722.7 ± 518.7 | 1133.9 ± 578.7 | 1186.6 ± 539.3 | 994.8 ± 400.2 |
| MDA5 (-) A/S-ILD, n = 13 | 202.3 ± 153.1 | 431.2 ± 300.2 | 710 ± 553.7 | 1264.6 ± 767.6 | 1406.9 ± 497.0 | 1143.5 ± 520.8 |
| p value | 0.62 | 0.65 | 0.95 | 0.58 | 0.25 | 0.36 |
| **T wave (μV)** |  |  |  |  |  |  |
| MDA5(-) non ILD, n = 22 | 2.0 ± 106.3 | 344.1 ± 201.5 | 427.5 ± 251.1 | 385.2 ± 223.6 | 311.6 ± 171.1 | 226.1 ± 124.0 |
| MDA5 (-) A/S-ILD, n = 13 | 36.5 ± 130.7 | 359.6 ± 267.9 | 523.8 ± 269.0 | 486.2 ± 231.1 | 380.8 ± 186.9 | 276.5 ± 158.9 |
| p value | 0.41 | 0.85 | 0.31 | 0.22 | 0.29 | 0.32 |
| **T/R ratio** |  |  |  |  |  |  |
| MDA5 (-) non ILD, n = 22 | -0.15 ± 0.67 | 0.68 ± 0.40 | 0.79 ± 0.74 | 0.35 ± 0.22 | 0.36 ± 0.45 | 0.26 ± 0.17 |
| MDA5 (-) A/S-ILD, n = 13 | -0.27 ± 1.72 | 1.16 ± 0.98 | 0.93 ± 0.65 | 0.57 ± 0.41 | 0.29 ± 0.14 | 0.26 ± 0.13 |
| p value | 0.80 | 0.08 | 0.61 | 0.06 | 0.62 | 0.99 |

Statistical significance: p < 0.05; Interstitial lung disease, ILD; acute or subacute ILD, A/S-ILD

**Supplementary Table 3**. Comparison of ECG parameters between the anti-MDA5 (+) ILD and anti-MDA5 (-) ILD groups in the active phase

| ECG | **HR　(bpm)** | **QT Int (ms)** |  |  |  |  |
| --- | --- | --- | --- | --- | --- | --- |
| MDA5 (+) ILD n = 21 | 79.5 ± 13.1 | 369.9 ± 30.4 |  |  |  |  |
| MDA5 (-) ILD n = 20 | 80.8 ± 14.0 | 369.4 ± 21.1 |  |  |  |  |
| p value | 0.78 | 0.95 |  |  |  |  |
|  |  |  |  |  |  |  |
| **R wave (μV)** | **Ⅰ** | **Ⅱ** | **Ⅲ** | **aVR** | **aVL** | **aVF** |
| MDA5 (+) ILD n = 21 | 554.8 ± 266.0 | 757.1 ± 293.1 | 339.8v313.1 | 61.4 ± 62.5 | 284.5 ± 241.7 | 522.1 ± 299.5 |
| MDA5 (-) ILD n = 20 | 491.0 ± 301.8 | 826.5 ± 428.3 | 584.3±469.9 | 53.5 ± 68.4 | 295.0 ± 296.1 | 690.0 ± 438.4 |
| p value | 0.49 | 0.56 | 0.06 | 0.71 | 0.90 | 0.17 |
| **T wave (μV)** |  |  |  |  |  |  |
| MDA5 (+) ILD n = 21 | 85.0 ± 43.0 | 113.1 ± 84.8 | 34.3 ± 72.4 | -97.4 ± 57.6 | 27.9 ± 40.8 | 65.5 ± 78.9 |
| MDA5 (-) ILD n = 20 | 123.0 ± 66.9 | 207.0 ± 107.1 | 89.3 ± 101.8 | －164.0 ± 76.1 | 20.8 ± 67.8 | 150.0 ± 92.9 |
| p value | 0.04 | <0.01 | 0.06 | <0.01 | 0.69 | <0.01 |
| **T/R ratio** |  |  |  |  |  |  |
| MDA5 (+) ILD n = 21 | 0.21 ± 0.19 | 0.16 ± 0.16 | 0.09 ± 0.82 | －1.98 ± 1.51 | 0.04 ± 0.65 | 0.12 ± 0.19 |
| MDA5 (-) ILD n = 20 | 0.50 ± 0.73 | 0.33 ± 0.24 | 0.31 ± 0.62 | －3.72 ± 2.36 | 0.03 ± 0.69 | 0.33 ± 0.28 |
| p value | 0.10 | 0.01 | 0.36 | 0.02 | 0.97 | <0.01 |
|  |  |  |  |  |  |  |
| **R wave (μV)** | **V1** | **V2** | **V3** | **V4** | **V5** | **V6** |
| MDA5 (+) ILD n = 21 | 168.6 ± 102.8 | 386.4 ± 212.8 | 596.2 ± 342.2 | 1206.0 ± 601.0 | 1627.9 ± 563.9 | 1286.2 ± 387.8 |
| MDA5 (-) ILD n = 20 | 202.0 ± 161.2 | 469.8 ± 323.7 | 727.5 ± 505.6 | 1320.5 ± 734.1 | 1513.8 ± 491.1 | 1202.5 ± 498.5 |
| p value | 0.44 | 0.35 | 0.35 | 0.60 | 0.51 | 0.56 |
| **T wave (μV)** |  |  |  |  |  |  |
| MDA5 (+) ILD n = 21 | 44.3 ± 116.4 | 286.7 ± 195.6 | 315.0 ± 206.7 | 238.6 ± 192.0 | 159.8 ± 145.7 | 114.3 ± 117.1 |
| MDA5 (-) ILD n = 20 | 56.3 ± 118.2 | 356.3 ± 242.5 | 479.3 ± 248.0 | 428.2 ± 227.7 | 321.8 ± 190.8 | 233.3 ± 151.7 |
| p value | 0.75 | 0.33 | 0.03 | <0.01 | <0.01 | <0.01 |
| **T/R ratio** |  |  |  |  |  |  |
| MDA5 (+) ILD n = 21 | 0.30 ± 0.86 | 0.79 ± 0.64 | 0.69 ± 0.62 | 0.28 ± 0.34 | 0.12 ± 0.13 | 0.11 ± 0.11 |
| MDA5 (-) ILD n = 20 | 0.24 ± 2.00 | 1.23 ± 1.36 | 0.87 ± 0.64 | 0.49 ± 0.40 | 0.24 ± 0.15 | 0.22 ± 0.13 |
| p value | 0.90 | 0.21 | 0.38 | 0.09 | <0.01 | <0.01 |

Statistical significance: p < 0.05; Interstitial lung disease, ILD

**Supplementary Table 4**. Comparison of the ECG parameters between the anti-MDA5 (+) A/S-ILD and anti-MDA5 (-) A/S-ILD groups in the active phase

| ECG | **HR　(bpm)** | **QT Int (ms)** |  |  |  |  |
| --- | --- | --- | --- | --- | --- | --- |
| MDA5 (+) A/S-ILD, n = 18 | 80.6 ± 12.3 | 370.6 ± 31.7 |  |  |  |  |
| MDA5 (-) A/S-ILD, n = 13 | 78.7 ± 13.8 | 371.4 ± 16.9 |  |  |  |  |
| p value | 0.70 | 0.94 |  |  |  |  |
|  |  |  |  |  |  |  |
| **R wave (μV)** | **Ⅰ** | **Ⅱ** | **Ⅲ** | **aVR** | **aVL** | **aVF** |
| MDA5 (+) A/S-ILD, n = 18 | 555.6 ± 257.0 | 762.2 ± 308.8 | 355.6 ± 330.8 | 61.1 ± 63.9 | 285.8 ± 241.5 | 530.0 ± 320.6 |
| MDA5 (-) A/S-ILD, n = 13 | 452.3 ± 261.0 | 831.9 ± 435.7 | 661.2 ± 500.9 | 53.5 ± 63.2 | 298.1 ± 322.6 | 748.5 ± 453.3 |
| p value | 0.30 | 0.62 | 0.06 | 0.75 | 0.91 | 0.14 |
| **T wave (μV)** |  |  |  |  |  |  |
| MDA5 (+) A/S-ILD, n = 18 | 79.7 ± 43.8 | 100.0 ± 76.0 | 26.1 ± 68.7 | －88.0 ± 52.2 | 30.8 ± 41.2 | 56.1 ± 70.6 |
| MDA5 (-) A/S-ILD, n = 13 | 137.3 ± 77.8 | 224.2 ± 109.9 | 87.7 ± 114.6 | －181.5 ± 78.8 | 30.8 ± 77.5 | 157.3 ± 100.9 |
| p value | 0.02 | <0.01 | 0.08 | <0.01 | 0.99 | <0.01 |
| **T/R ratio** |  |  |  |  |  |  |
| MDA5 (+) A/S-ILD, n = 18 | 0.19 ± 0.16 | 0.13 ± 0.12 | -0.01 ± 0.82 | －1.96 ± 1.60 | 0.06 ± 0.68 | 0.10 ± 0.17 |
| MDA5 (-) A/S-ILD, n = 13 | 0.53 ± 0.80 | 0.35 ± 0.25 | 0.34 ± 0.75 | －3.80 ± 2.21 | 0.09 ± 0.78 | 0.33 ± 0.30 |
| p value | 0.10 | <0.01 | 0.26 | 0.03 | 0.91 | 0.01 |
|  |  |  |  |  |  |  |
| **R wave (μV)** | **V1** | **V2** | **V3** | **V4** | **V5** | **V6** |
| MDA5 (+) A/S-ILD, n = 18 | 173.1 ± 101.8 | 412.2 ± 205.9 | 658.0 ± 323.5 | 1246.4 ± 621.3 | 1655.0 ± 574.8 | 1286.9 ± 403.0 |
| MDA5 (-) A/S-ILD, n = 13 | 202.3 ± 153.1 | 431.2 ± 300.2 | 710 ± 553.7 | 1264.6 ± 767.6 | 1406.9 ± 497.0 | 1143.5 ± 520.8 |
| p value | 0.54 | 0.84 | 0.75 | 0.94 | 0.24 | 0.41 |
| **T wave (μV)** |  |  |  |  |  |  |
| MDA5 (+) A/S-ILD, n = 18 | 53.6 ± 116.5 | 289.7 ± 208.8 | 317.2 ± 219.9 | 233.6 ± 201.9 | 148.3 ± 147.0 | 99.1 ± 111.9 |
| MDA5 (-) A/S-ILD, n = 13 | 36.5 ± 130.7 | 359.6 ± 267.9 | 523.8 ± 269.0 | 486.2 ± 231.1 | 380.8 ± 186.9 | 276.5 ± 158.9 |
| p value | 0.71 | 0.44 | 0.03 | <0.01 | <0.01 | <0.01 |
| **T/R ratio** |  |  |  |  |  |  |
| MDA5 (+) A/S-ILD, n = 18 | 0.31 ± 0.89 | 0.78 ± 0.65 | 0.64 ± 0.59 | 0.27 ± 0.35 | 0.11 ± 0.13 | 0.09 ± 0.10 |
| MDA5 (-) A/S-ILD, n = 13 | -0.27 ± 1.72 | 1.16 ± 0.98 | 0.93 ± 0.65 | 0.57 ± 0.41 | 0.29 ± 0.14 | 0.26 ± 0.13 |
| p value | 0.26 | 0.22 | 0.25 | 0.05 | <0.01 | <0.01 |

Statistical significance: p < 0.05; Interstitial lung disease, ILD; acute or subacute ILD, A/S-ILD

**Supplementary Table 5.** UCG parameters in the remission phase

| UCG | MDA5 (+)  n = 11 | MDA5 (-)  n = 15 |
| --- | --- | --- |
| LVEF (%) | 70.0 ± 9.5 | 61.9 ± 14.7 |
| LVDd (mm) | 41.3 ± 4.1 | 43.9 ± 5.8 |
| LVDs (mm) | 25.1 ± 5.0 | 27.9 ± 7.0 |
| IVSTd (mm) | 9.8 ± 1.8 | 9.4 ± 2.0 |
| PWTd (mm) | 9.6 ± 1.6 | 9.4 ± 2.0 |
| LADs (mm) | 34.5 ± 7.0 | 34.1 ± 6.1 |
| E wave (m/sec) | 0.60 ± 0.14 | 0.68 ± 0.16 |
| A wave (m/sec) | 0.62 ± 0.22 | 0.74 ± 0.14 |
| E/A | 1.2 ± 0.9 | 0.97 ± 038 |
| DcT (msec) | 228.8 ± 66.6 | 197.2 ± 49.4 |
| Sep E/e' | 10.7 ± 5.9 | 10.4 ± 2.6 |
| Sep e’ (cm/sec) | 6.1 ± 1.4 | 6.7 ± 1.5 |
| Sep a’ (cm/sec) | 9.4v2.9 | 9.6 ± 1.9 |
